# Factors associated with changes in health-related quality of life in children with cystic fibrosis during 1-year follow-up

**DOI:** 10.1007/s00431-017-2928-6

**Published:** 2017-06-09

**Authors:** Marieke van Horck, Bjorn Winkens, Geertjan Wesseling, Karin de Winter-de Groot, Ilja de Vreede, Quirijn Jöbsis, Edward Dompeling

**Affiliations:** 1 Department of Paediatric Respiratory Medicine, School for Public Health and Primary Health Care (CAPHRI), Maastricht University Medical Centre + (MUMC+), Maastricht, The Netherlands; 2 Department of Methodology and Statistics, CAPHRI, MUMC+, Maastricht, The Netherlands; 3 Department of Respiratory Medicine, CAPHRI, MUMC+, Maastricht, The Netherlands; 4 Department of Paediatric Respiratory Medicine, Wilhelmina Children’s Hospital, University Medical Centre Utrecht (UMCU), Utrecht, The Netherlands; 5 Department of Paediatric Respiratory Medicine, Leiden University Medical Centre (LUMC), Leiden, The Netherlands

**Keywords:** Cystic fibrosis (CF), Quality of life, Children, Longitudinal design

## Abstract

There are limited data on health-related quality of life (HRQoL) changes over time in children with cystic fibrosis (CF). We investigated associations between clinical and treatment variables with changes in HRQoL during 1 year. Forty-nine children with CF aged 6–18 years were followed in this multicentre, observational cohort study during 1 year. HRQoL was measured by the validated disease specific cystic fibrosis questionnaire-revised (CFQ-R). The CFQ-R total score as well as most domain scores improved significantly (8.0 points and [3.3–31.7] points respectively) during the one-year follow-up. Age at baseline demonstrated a strong longitudinal association with the change of CFQ-R total score (2.853 points decrease of CFQ-R total score per year increase in age) and several domain scores. Below 12 years of age, CFQ-R total score improved in most children, whereas a deterioration was observed in most children above 12 years. The number of PEx was associated with an increase of treatment burden score (4.466 points decrease per extra PEx).

*Conclusion*: In the group as a whole, HRQoL improved significantly over time. However, changes over time were significantly influenced by age: below 12 years of age, HRQoL improved in most patients whereas a deterioration was observed in most children >12 years. Strategies how to preserve or ideally to improve HRQoL in adolescence should be developed.

**Table:** 

**What is known:** *• Quality of life in patient with* CF *is diminished* *• Although* CF *is a chronic disease, longitudinal data on QoL in children with* CF *are scarce.*
**What** **is new:** *• Below 12 years of age, quality of life improved in most children during the 1-year follow-up whereas a deterioration in quality of life was observed in most children above 12 years.* *• the treatment burden score of QoL correlated with the exacerbation rate*

## Introduction

Survival in cystic fibrosis (CF) has improved drastically over the past decades but is also accompanied by more CF-related comorbidities and a greater treatment burden, which may impact quality of life [17]. Quality of life in a chronic disease like CF is not only of major importance to patients but also to the treating physicians. Fortunately, in the management of CF, there is an increasing focus on survival in combination with a good health-related quality of life (HRQoL) [18]. Moreover, HRQoL is increasingly used as a patient-related-outcome measure for clinical trials in patients with CF [8].Clinicians should be able to assess both the medical benefit and the contribution to quality of life of (new) treatments for each individual patient [18]. Hence, it is important to identify factors associated with HRQoL, so clinicians can focus on prioritizing and optimizing management of factors that impact it at the most [9]. The cystic fibrosis questionnaire-revised (CFQ-R) is a disease specific, validated HRQoL measure [14].

Most research on quality of life in CF is performed in adults and has a cross-sectional study design, whereas the majority of longitudinal studies on HRQoL was conducted in clinical trials of CF therapies. Longitudinal data on HRQoL in non-intervention studies with children are currently lacking. Several cross-sectional studies showed that age, gender, body mass index (BMI), pulmonary exacerbations (PEx), and forced expiratory volume in 1 s (FEV_1_) appear to impact HRQoL as measured by the CFQ-R [4, 10, 11, 16].

Sawicki et al. demonstrated significant associations between respiratory symptoms and CFQ-R respiratory symptom scores and between weight and scores on CFQ-R nutritional health domains in children and adults during 9–15 months of follow-up [20].

In this prospective observational study during 1 year, we investigated associations between clinical and treatment variables with changes in CFQ-R scores in children with CF. We assumed that (1) pulmonary exacerbations and lung function decline are related to worsening of CFQ-R respiratory health domains; (2) the number of medications/inhalation therapies are associated with higher CFQ-R treatment burden scores.

## Methods

### Study design and patients

Children with CF aged 5 to 19 years were included in this 1-year, multicentre, observational cohort study (clinicaltrial.gov NCT01241890). Children were recruited from three CF centres in the Netherlands (Maastricht, Utrecht, and Amsterdam).

CF was defined as the presence of characteristic clinical features (persistent pulmonary symptoms, meconium ileus, failure to thrive, and steatorrhea) in combination with an abnormal sweat test (chloride >60 mM) and/or two CF mutations [21]. Exclusion criteria were (1) severe cardiac abnormalities; (2) mental disability; (3) no technically adequate performance of measurements; (4) on waiting list for lung transplantation; (5) children colonized with *Burkholderia cepacia or Methicillin-resistant Staphylococcus aureus*; (6) participation in an intervention trial.

Children with *B. cepacia* or *MRSA* were excluded as part of the study (not reported here) included breath measurements (exhaled volatile organic compounds and cytokines in exhaled breath condensate) which have a risk of cross-infection of other patients by the breath sampling equipment. Ethical approval was obtained from the Medical Ethical Committee of the Maastricht University Medical Centre. Informed consent was signed by all parents, and by children aged 12 years and over.

### Study parameters

For the period of 1 year, all children attended regular clinical visits every 2 months. Demographic information and clinical characteristics (pancreatic insufficiency [use of pancreatic enzymes], BMI *z* scores, and colonization with *Pseudomonas aeruginosa*) were collected at inclusion. During each clinical visit, changes in treatment/medications were reported (total number of medications, number of inhalation therapies [such as DNA-se, hypertonic saline, or antibiotic], and use of insulin). Besides, *dynamic lung function parameters* (Masterscreen Pneumo, Carefusion, Houten, The Netherlands) were assessed according to international standards [13]. Recorded parameters were FEV_1_, forced vital capacity (FVC), and maximum expiratory flow at 50% of FVC (MEF_50_), all expressed as absolute rate and as percentage of the predicted normal value. Children and their parents were asked to fill in the *CFQ-R questionnaire* three times during the study, at inclusion, after 6 months and after 1 year of follow-up. We defined change in CFQ-R scores during 1 year as main outcome measure (excluding CFQ-R scores filled in after 6 months). We used the translated and validated Dutch CFQ-R questionnaires appropriate for the different age groups [11]: 6–13 years (child interview), 13–14 years of age (child self-report), and ≥14 years (adolescents). Furthermore, parents of children aged 6–13 years reported on a special parent version. The CFQ-R consists of 35–50 items divided into 7–9 domains (depending on age group): physical functioning, energy and well-being, emotional state, social limitations, role limitations, body image, eating disturbances, treatment burden, and embarrassment. Moreover, overall health perception and three symptom scales are included: respiratory, digestive, and weight. Items require either a frequency response on a 4-point scale (‘all the time’ to ‘never’), a difficulty rating on a 4-point scale (‘a lot of difficulty’ to ‘no difficulty’), a true–false rating on a 4-point scale, or the selection of a statement that describes the patient (on a 3- or 4-point scale). The scores range from 0 to 100 with higher scores corresponding to higher quality of life. Only for the respiratory symptom domain, a minimal clinically important difference (MCID), the smallest clinically relevant change a patient can detect, of 4.0 points (in stable patients) is determined [15]. For the analysis of the separate domains, only those completed by all age groups were included (excluding energy and well-being, role limitations, embarrassment, and the weight symptom scale). The main outcome was changed in CFQ-R total and domain scores in 1 year.

PEx were defined by courses of therapeutic antibiotics (intravenous and oral) prescribed by the responsible paediatric pulmonologists, considering the clinical symptoms as an expression of a PEx. PEx were treated according to the Dutch Central Guidance Committee (CBO) guideline [5], which resembles European [21] and American CF guidelines [7].

### Statistical analysis

Descriptive statistics of the baseline characteristics were expressed as mean (standard deviation [SD]) or as median (interquartile range (IQR), i.e. 25th–75th percentile) for numerical variables, and as number (percentage) for categorical variables. Differences between those children with one or more missing covariate and/or outcome value and those included in the complete case analysis (CCA) were tested using independent-samples *t* test or Mann-Whitney *U* test for numerical variables and chi-square test for categorical variables.

To analyse the associations between demographic and clinical characteristics and changes in CFQ-R scores (*T* = 12 minus *T* = 0), univariable and multivariable linear regression models were used. Dependent variables for the models were changed in CFQ-R total score and CFQ-R domain scores. All covariates (demographic and clinical characteristics) with a *p* value ≤0.20 from the univariable analyses were entered in the multivariable analysis.

To deal with missing data, 50 complete datasets were created using multiple imputation (MI). The maximum number of iterations was set equal to 20, where convergence was checked by inspecting the trace lines. The missing covariate and outcome values were imputed using all other variables as predictors, where the outcome variable was included to impute missing covariate values. The CCA, in which the patients with one or more missing covariate values were excluded from the analysis, was also performed and served as a sensitivity analysis.

Data were analysed with IBM SPSS Statistics for Windows (version 22.0. Armonk, NY). The multiple imputation part was performed using the MICE package in R (version 3.2.3) [23].

A post-hoc power analysis showed that with 49 cases, correlations between changes in CFQ-R scores and clinical variables of ≥0.30 can be detected with a power of 0.80 and a significance level *α* of 0.05, which was evaluated as sufficiently accurate for the purpose of this clinical study.

## Results

Forty-nine patients participated in this 1-year prospective observational study. For the analysis of HRQoL, 39 of them (80%) had no missing values (covariates and outcome parameters) and were included in the CCA. Table 1 shows the baseline demographic and clinical characteristics of all 49 children and of the two subgroups, i.e. those without CCA and those with any missing covariates or outcome variables (‘missing’). There were no significant differences between those in the CCA and those in the ‘missing’ group.

**Table 1:** Baseline characteristics of the 49 children

Characteristic	Total cohort (*n* = 49)	CCA (*n* = 39)	‘missing’ (*n* = 10)	*p* value
Age, mean (SD)	10.3 (3.6)	10.3 (3.2)	10.3 (4.9)	0.984
Male sex, *N* (%)	31 (63.3)	26 (66.7)	5 (50.0)	0.329
*Pseudomonas Aeruginosa *at inclusion^a^, *N* (%)	15 (30.6)	12 (30.8)	3 (30.0)	0.962
Pancreatic insufficiency, *N* (%)	32 (65.3)	25 (64.1)	7 (70.0)	0.727
CFRD	4 (8.2)	4 (10.3)	0 (0.0)	0.291
Pathology of upper respiratory tract	21 (42.9)	16 (41.0)	5 (50.0)	0.609
FEV_1_% of predicted value, mean (SD)	87.4 (18.1)	87.1 (17.5)	88.6 (21.2)	0.816
BMI *z* score, mean (SD)	0.14 (0.83)	0.17 (0.81)	0.05 (0.94)	0.675
Number of PEx, median (IQR)	2.0 (1.0–4.0)	2.0 (1.0–4.0)	1.0 (0.0–3.3)	0.384
Number of medications^b^, median (IQR)	7.0 (5.0–9.0)	7.0 (5.0–9.0)	6.0 (4.8–7.5)	0.320
Number of inhalation therapies, median (IQR)	2.0 (1.0–2.0)	2.0 (1.0–3.0)	1.5 (1.0–2.0)	0.687

*BMI* body mass index, *CFRD* cystic fibrosis-related diabetes, *FEV*_*1*_ forced expiratory volume in 1 s, *FVC* forced vital capacity, *PEx* pulmonary exacerbations

*p < 0.05

^a^Treated because of the presence in sputum

^b^Total number of medications minus total number of inhalation therapies

### Course of CFQ-R scores

Table 2 shows the mean CFQ-R total score and domain scores at *T* = 0, *T* = 12, and the change from baseline during follow-up. All scores improved significantly during the year except digestive symptoms. The mean total CFQ-R score was 68.6 at *T* = 0 and 76.6 at *T* = 12, a significant improvement of 8.0 points. Almost all domain scores also improved significantly, from 3.3 points (respiratory symptoms domain) to 31.7 points (physical functioning domain).

**Table 2:** CFQ-R total and domain scores at *T* = 0 and *T* = 12 and change over time

Characteristic	*T* = 0, mean (SD)	*T* = 12, mean (SD)	Change (*T* = 12–*T* = 0), mean (SD)	*p* value
CFQ-R total score, mean (SD)	68.6 (11.1)	76.6 (9.8)	8.0 (16.3)	<0.001*
CFQ-R physical functioning, mean (SD)	48.5 (31.8)	80.2 (16.6)	31.7 (41.3)	<0.001*
CFQ-R emotional functioning, mean (SD)	76.3 (12.0)	82.1 (11.0)	5.8 (11.2)	<0.001*
CFQ-R social functioning, mean (SD)	61.5 (17.0)	73.3 (17.8)	11.8 (27.5)	<0.001*
CFQ-R body image, mean (SD)	82.0 (21.2)	91.6 (10.8)	9.6 (19.2)	<0.001*
CFQ-R eating disturbances, mean (SD)	70.5 (17.9)	76.5 (26.5)	6.0 (24.0)	<0.001*
CFQ-R treatment burden, mean (SD)	61.6 (20.6)	69.6 (24.7)	8.0 (36.6)	<0.001*
CFQ-R respiratory symptoms, mean (SD)	79.3 (14.6)	82.6 (12.0)	3.3 (16.0)	<0.001*
CFQ-R digestive symptoms, mean (SD)	69.5 (23.2)	68.7 (24.6)	−0.8 (32.9)	0.211
FEV_1_% of predicted value, mean (SD)	87.1 (17.5)	87.1 (19.0)	−0.03 (10.8)	0.987

*CFQ-R* cystic fibrosis questionnaire-revised

*p < 0.05

### Associations with change in CFQ-R total score

The MI analyses showed that, after correction for the other covariates, only age at baseline had a strong longitudinal association with change of CFQ-R total score in 1 year (Table 3). The estimate (B) indicates that with every year a child gets older, the mean change from baseline in CFQ-R total score decreased with 2.853. The relationship between PEx and the change in CFQ-R total score was of borderline significance (*p* = 0.065). Sex, BMI *z* score, pancreas insufficiency at *T* = 0, number of medications during the study, CFRD at *T* = 0, pathology of the upper respiratory tract at *T* = 0, and CFQ total score at *T* = 0 were not included in the MI (all *p* values from univariable analyses were ≥0.20). Similar results were found in de CCA. No correlation between the change in HRQoL and the change in FEV1 was found (Pearson correlation coefficient *r* = 0.036, *p* = 0.83).

**Table 3:** Associations between demographic/clinical variables and changes from baseline in CFQ-R total score using multivariable linear regression analysis

Covariate	B (CI)	*p* value
Age at *T* = 0	−2.853 (−3.915, −1.791)	<0.001*
FEV_1_% pred at *T* = 0	0.030 (−0.197, 0.256)	0.797
Number of PEx during study	−1.839 (−3.790, 0.113)	0.065
Colonization with *Pseudonomas aeruginosa* at *T* = 0^a^	−5.527 (−13.985, 2.931)	0.200
Number of inhalation therapies during study	0.186 (3.532, 3.903)	0.922

*FEV*_*1*_ *%pred* forced expiratory volume in 1 s as percentage of predicted, *PEx* pulmonary exacerbations

*p < 0.05

^a^Treated because of presence in sputum

**Fig. 1:**
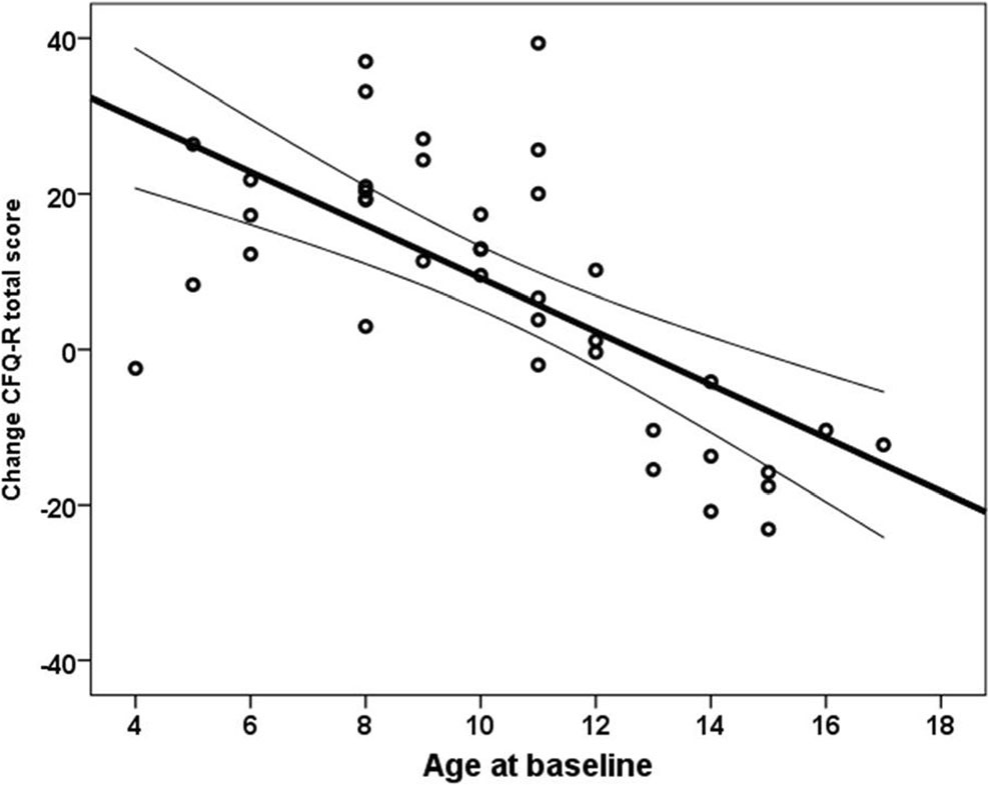
Association between age at baseline and change in CFQ-R total scores (regression line and 95%CI)

Figure 1 shows the association between age at baseline and CFQ-R total scores of the CCA.

### Associations with change in separate CFQ-R domain scores

In the multivariable regression models of the separate CFQ-R domains, increase in age was consistently associated with worsening of physical functioning and social functioning domain scores, treatment burden score, and respiratory symptoms score (Table 4). Of the other covariates, the number of PEx was independently associated with increasing treatment burden score, whereas the total number of (non-inhalation) therapies was associated with worsening of physical functioning domain score. In contrast, multivariable regression models did not show significant associations with any covariate and changes in the domains of emotional functioning, eating disturbances, body image, and digestive symptoms.

**Table 4:** Associations between demographic/clinical variables and changes in CFQ-R domains (only results of multivariable analyses are shown)

Covariate	Physical functioning (B, CI, *p* value)	Social functioning (B, CI, *p* value)	Treatment burden (B, CI, *p* value)	Respiratory symptoms (B, CI, *p* value)
Age at *T* = 0	−3.766 (−6.565, −0.967) 0.008*	−4.188 (−6.150, −2.226) <0.001*	−6.995 (−9.401, −4.589) <0.001*	−1.450 (−2.859, −0.41) 0.044*
BMI *z* score at *T* = 0	0.102 (−10.576, 10.780) 0.985	1.531 (−7.047, 10.137) 0.727	*a)*	−4.280 (−10.301, 1.742) 0.163
FEV_1_% pred at *T* = 0	0.120 (−0.400, 0.640) 0.650	*a)*	*a)*	*a)*
Number of PEx during study	−3.819 (−8.092, 0.453) 0.080	−2.352 (−5.748, 1.044) 0.175	−4.466 (−8.821, −0.111) 0.044*	*a)*
Pancreas insufficiency at *T* = 0		−11.219 (−24.839, 2.402) 0.106	*a)*	*a)*
Number of medications during study^a^	−4.097 (−7.254, −0.940) 0.011*	*a)*	*a)*	*a)*
Number of inhalation therapies during study	1.079 (−7.107, 9.265) 0.796	*a)*	0.633 (−6.308, 7.574) 0.858	*a)*
CFRD at *T* = 0	−7.219 (−38.890, 24.452) 0.655	*a)*	*a)*	*a)*
Pathology of upper respiratory tract at *T* = 0	*a)*	−7.758 (−21.648, 6.133) 0.274	*a)*	9.074 (−0.271, 18.419) 0.057
CFQ-R domain score at *T* = 0	−1.396 (−2.080, −0.711) <0.001*	*a)*	*a)*	−0.297 (−0.701, 0.107) 0.149

*a)* not included in multivariable analyses (*p* values from univariable analyses were ≥0.20)

*BMI* body mass index, *CFRD* cystic fibrosis-related diabetes, *CFQ* cystic fibrosis questionnaire-revised, *FEV*_*1*_ *% pred* forced expiratory volume in 1 s as percentage of predicted, *PEx* pulmonary exacerbations

*p < 0.05

^a^Total number of medications minus total number of inhalation therapies

## Discussion

In this 1-year observational study, we investigated longitudinal associations between clinical and treatment variables with changes in HRQoL in children with CF. Overall, CFQ-R total score and all domain scores, except the digestive symptom score, improved significantly during 1 year. An older age was the most important determinant of a deterioration in CFQ-R total score and in several domain scores, whereas the CFQ-R total score improved in most children <12 years. Furthermore, PEx were significantly related to an increase in treatment burden score, and the total number of non-inhalation therapies correlated with a deterioration of the physical functioning domain score during 1 year.

To our knowledge, this is the first longitudinal non-intervention study in children with CF to investigate the impact of clinical and treatment variables on change in HRQoL. We found significant improvements in CFQ-R total scores as well as most domain scores. We speculate that merely participating in an intensive study like the present one improves HRQoL because of the extra attention that patients get, the extra contacts with the nurses and physicians of the CF team, and maybe also a better treatment adherence during the study. An alternative explanation for the main findings (Fig. 1) may be a better coping with the disease in the course of years after birth (during school age) and the problems related to CF in teenagers. It is interesting to perform in depth/focus interviews with parents and children with CF to get a better idea about underlying reasons for changes in HRQoL. The drop in QoL in children of 12 years and over maybe the consequence of the increasing disease severity and treatment intensity, as reflected by the scores of the treatment burden domain and the respiratory symptom domain. Our results are in accordance with Abbott et al. who showed a significant deterioration of all CFQ-R domain scores in adolescents and adults during a decade of follow-up (−6.0 to −15.9 points) [2]. In children (6–13 years of age), Sawicki et al. found a significant increase in respiratory symptom score over 1 year, but no significant changes in the other domains [20]. In adults, Dill et al. found that although individual variation exists, overall, there was no significant change in physical HR-QoL during 21 months of follow-up [6]. However, there were significant time trends in three psychosocial domains: treatment burden (improvement), emotional functioning (improvement), and social functioning (deterioration) [6].

Others also found an effect of age on HRQoL. Hegarty et al. demonstrated that children aged 6–13 years scored significantly better than those aged 14–18 for ‘emotional state’, ‘body image’, and ‘treatment burden’ [10]. Moreover, a large European study in healthy children found a gradual decrease in HRQoL from childhood into adolescence [12]. Abbott et al. evaluated the relationship between demographic and clinical variables and HRQoL during a 12-year period in adolescents and adults. In this study, the importance of advancing age as predictor of HRQoL was confirmed [3]. Especially in children with CF, not much is known about ‘normal’ CFQ-R scores. The HRQoL Outcomes Group warns about the difficulty of interpreting changes in CFQ-R scores; there is no minimal clinically important difference known for the CFQ-R scores except for the respiratory symptom domain [1, 15]. Tibosch et al. found that the majority of healthy children do not reach maximum scores on many domains of the CFQ-R [22]. Moreover, they found a heterogeneity of scores between the different domains, and there were differences according to age [22]. This implies normal psychosocial development, and puberty should be taken into account when interpreting HRQoL especially in childhood and adolescence [22].

We hypothesised that pulmonary exacerbations and lung function decline would be related to a deterioration of CFQ-R respiratory health domains. PEx were significantly related to an increase in treatment burden score but not with respiratory health domains or other domains in our study. Dill et al. showed that PEx were associated with lower CFQ-R scores of all domains in adults [6]. In our study, there was a positive time-trend in HRQoL; this might be the reason why we did not find an effect of PEx. Besides, we found no significant influence of FEV_1_% pred on any CFQ-R domain. A longitudinal study during 12 years showed that a decrease in lung function was associated with a decrease in HRQoL in adolescents and adults [2]. We did not find this association which could be due to the relatively preserved and stable FEV_1_% pred during follow-up of only 1 year.

Furthermore, we speculated that CFQ-R treatment burden scores would be influenced by the number of (inhalation/nebulisation) therapies. However, we found no significant association between number of medications (inhalation as well as non-inhalation) and treatment burden scores. Ziaian et al. also found no significant relationship between treatment time (dependent on number of treatments) and HRQoL (measured with the Child Health Questionnaire, a non-disease specific tool) of children with CF [24]. Moreover, Sawicki et al. investigated treatment complexity (based on daily frequency, duration, and ease of administration of chronic medications) and found that changes in treatment complexity were not associated with worsening treatment burden scores in children and adolescents [20]. These findings are in contrast with a study in adults, where using two or more nebulized medications was significantly associated with increased treatment burden [19]. However, we found that PEx were significantly related to an increase in treatment burden score, which probably implies that a temporary increase in number of medications and/or treatment time due to the exacerbation does matter.

The strengths of our study are the longitudinal study design, the focus on children only, and assessing HRQoL in a non-interventional study. Although the power analysis showed that clinically meaningful correlations could be detected with this relatively small sample size, it is still a limitation, for example, subanalyses for different age groups were not possible. Another limitation is the presence of missing data, in 10 of the 49 children, one or more covariate or outcome variable was missing. We used multiple imputations to overcome this problem, which assumes the data to be missing at random. In this study group, the percentage of children with pancreas insufficiency (65%) was lower than in the national Dutch cohort (80–90% pancreas insufficiency).The results of this study may not hold for children with *B. cepacia* or *MRSA* patients as they were not included in this study. Although statistically significant improvements were observed in most QoL scores over time, these changes may not be clinically meaningful as MCID of most QoL scores is unknown.

The clinical implication of our study is that age and puberty have impact on HRQoL. Longitudinal changes in HRQoL in children with CF are relevant, and HRQoL may therefore at least be included in the annual clinical evaluation of patients. It is worthwhile to repeat the study in a larger cohort and to assess more background information about experiencing a better or worse HRQoL, for instance by focus interviews. Besides, strategies to improve HRQoL in children with CF are very important, particularly in teenagers.

In conclusion, an older age is by far the most important variable related to a deterioration in HRQoL in children with CF. Besides, PEx and number of inhalation therapies were related to treatment burden and physical functioning. This means physicians should focus on HRQoL in adolescents and develop strategies on how to preserve or even improve HRQoL in this age group.

## Abbreviations

BMI: Body mass index
CCA: The complete case analysis
CF: Cystic fibrosis
CFRD: Cystic fibrosis-related diabetes;
CFQ-R: Disease specific cystic fibrosis questionnaire-revised
FEV_1_: Forced expiratory volume in 1 s
FVC: Forced vital capacity;
HRQoL: Health-related quality of life
MI: Multiple imputation
PEx: Pulmonary exacerbations
QoL: Quality of life
